# Investigation of biases in convolutional neural networks for semantic segmentation using performance sensitivity analysis

**DOI:** 10.1016/j.zemedi.2021.11.004

**Published:** 2022-01-10

**Authors:** Daniel Güllmar, Nina Jacobsen, Andreas Deistung, Dagmar Timmann, Stefan Ropele, Jürgen R. Reichenbach

**Affiliations:** 1 Medical Physics Group, Institute of Diagnostic and Interventional Radiology, Jena University Hospital - Friedrich Schiller University Jena, Germany; 2 University Clinic and Outpatient Clinic for Radiology, Department for Radiation Medicine, University Hospital Halle (Saale), Germany; 3 Department of Neurology, University Hospital Essen, University of Duisburg-Essen, Essen, Germany; 4 Department of Neurology, Karl-Franzens University of Graz, Austria; 5 Michael Stifel Center Jena for Data-Driven and Simulation Science, Friedrich-Schiller-University Jena, Jena, Germany

**Keywords:** Sensitivity analysis, Semantic image segmentation, Convolutional neural network, Data augmentation

## Abstract

The application of deep neural networks for segmentation in medical imaging has gained substantial interest in recent years. In many cases, this variant of machine learning has been shown to outperform other conventional segmentation approaches. However, little is known about its general applicability. Especially the robustness against image modifications (e.g., intensity variations, contrast variations, spatial alignment) has hardly been investigated. Data augmentation is often used to compensate for sensitivity to such changes, although its effectiveness has not yet been studied. Therefore, the goal of this study was to systematically investigate the sensitivity to variations in input data with respect to segmentation of medical images using deep learning. This approach was tested with two publicly available segmentation frameworks (DeepMedic and TractSeg). In the case of DeepMedic, the performance was tested using ground truth data, while in the case of TractSeg, the STAPLE technique was employed. In both cases, sensitivity analysis revealed significant dependence of the segmentation performance on input variations. The effects of different data augmentation strategies were also shown, making this type of analysis a useful tool for selecting the right parameters for augmentation. The proposed analysis should be applied to any deep learning image segmentation approach, unless the assessment of sensitivity to input variations can be directly derived from the network.

## Introduction

The use of convolutional neural networks (CNN) to solve segmentation problems in magnetic resonance imaging (MRI) has gained wide acceptance due to their successful application [1], [2], [3], [4]. However, despite continuous improvements in both the network architecture and training schemes, there are still limitations due to the problem of data-imbalances [2], [5]. Although several CNNs have been proven to perform better than traditional atlas-based or statistical segmentation methods when trained and tested with data that have similar acquisition characteristics in terms of intensity ranges, contrast between tissue groups, resolution, and image orientation [2], [3], these networks tend to perform worse than traditional segmentation methods when tested with data sets that differ from the training sample. This restricts their general applicability [2]. For MRI data in particular, variations between training sample populations and the sample populations used for evaluation are to be expected, especially when data are acquired with different sequence parameters or with different scanners. If such expected variations are not accounted for, deep learning (DL) applications will be biased by the training sample population. One approach to overcome this bias is to enlarge the range of data variability in the training sample as CNN training progresses. This process is referred to as data augmentation and includes a wide range of techniques, all of which aim to expand the parameter space of the data of interest. With respect to MR images, the parameters could, for example, originate from different sequence configurations, different scanners, or variations in the subjects’ morphology. Among the most common methods of data augmentation to mimic such variations are modifications such as translation, flipping, rotation, scaling, or intensity manipulation of data sets [6], [7]. While these modifications improve CNN segmentation performance, they are limited to their respective parameter spaces, which can be challenging to determine. For this reason, more advanced methods using Generative Adversarial Networks (GANs) have gained popularity as they allow complex data synthesis and show significant performance improvements when exploring high dimensional data distributions [8], [9], [10], [11], [12], [13]. Drawbacks of data augmentation, including methods involving GANs, entail limitation to the feature space that has been presented to the networks during training. In fact, image synthesis using GANs may even enhance biases present in the training data set [14], [15]. Despite the wide range of existing solutions to improve CNN performance to successfully segment an expanded number of datasets, the impact of an implemented augmentation method is primarily evaluated based on accuracy measures obtained by single forward computations for a set of test samples [16]. Although improved CNN generalizability is then suggested by improved segmentation performance, the extent of these improvements is commonly not assessed. To address this issue, we take a simple approach to assessing the sensitivity of the network to a particular parameter, leading to an analysis strategy that can be used to describe the actual generalizability of the network and to visualize potential problems. We studied segmentation of the cerebellum based on structural T1-weighted (T1w) 3D MR images as well as segmentation of white matter tracts based on MR diffusion-weighted images as use cases with two different CNNs. We analyzed the sensitivity of both networks to intensity changes and rotation angles and compared CNNs trained with and without the use of the corresponding augmentation techniques.

## Material and methods

In the following, we give a brief general description of our methodology for analyzing the sensitivity of the network performance before presenting the two use cases.

### Performance sensitivity analysis

The strategy for assessing the sensitivity of segmentation results to modifications in the input data was to create and visualize a quantitative measure representing the sensitivity of a CNN's performance in relation to a specific parameter space. The analysis consists of overall three steps, and requires a trained neural network as well as at least one test data set with a ground truth volume available. In step 1, each instance of the test data is systematically modified based on a specific transfer function to manipulate the images with an arbitrary number of free variables. These variables, such as spatial rotation and scaling parameters or intensity changes, span an N-dimensional parameter space. All systematic modifications of the test data set are processed in step 2 using the trained neural network. In step 3, the segmentations and their corresponding ground truth volumes are compared using an evaluation metric and the result is plotted in an N-dimensional plot corresponding to the chosen parameter space. In this study, we restrict ourselves to the presentation of results of such analyses in two-dimensional parameter space, which can then be visualized as heat maps using the Sørensen-Dice Similarity Coefficient (DSC) [17] as the evaluation metric.

**Figure 1:**
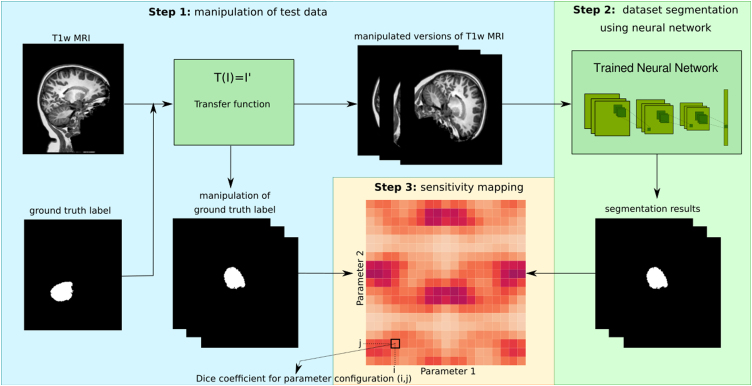
Diagram of the workflow of the network performance sensitivity analysis, illustrated for a rotation with two degrees of freedom (rotation around the x- and y-axis, respectively). The test data set, including an MR volume and its corresponding ground-truth label, is first modified within a chosen parameter range using a specified transfer function. The modified test data sets are subsequently fed into a trained neural network, which performs the segmentation. In the final step, the resulting CNN segmentations are compared to the corresponding ground truth label volumes using the performance metric and the values of the latter are displayed as a function of the two parameters as a heat map.

The DSC is given by

(1) $$DSC(v_{gt},V_{cnn})=\frac{2|V_{gt}\cap V_{cnn}|}{\left|V_{gt}|+|V_{cnn}\right|}$$

where *V*_*gt*_ represents the ground truth volume and *V*_*cnn*_ the segmentation result of the CNN. The DSC values range between 0 and 1, where 1 indicates complete overlap of the two segmentations. The workflow of described analysis is presented graphically in Figure 1. When interpreting such a graphical representation, in the optimal case (performance independent of the examined parameters) a homogeneous distribution of measured values with high (e.g. near 1.0 for the DICE score) or low (e.g. near 0.0 for a dissimilarity measure like SSE) amplitude is obtained. However, if there are visual patterns (e.g., segmentation performance depends on one or more parameters of the input data), this indicates a dependency between the investigated input parameter and segmentation performance, and then asks for the potential effects of data augmentation on this dependency to be investigated in more detail.

### Application case 1: segmentation of the cerebellum

**Data acquisition and preprocessing** – The training sample comprised 150 3D MRI datasets (130 healthy controls and 20 patients suffering from cerebellar atrophy), collected at the Essen University Hospital (EUH) on a 3 T MRI scanner with a voxel size of (0.96 × 0.96 × 1.0) mm^3^. Fat suppression was employed in 50% of the cases. Spatially labeled volumes of the cerebellum including the brainstem were obtained using the SUIT toolbox [18], followed by manual correction if necessary. To include only cerebellar white and gray matter, the brainstem volume was excluded from the SUIT labels by using the brainstem segmentation result computed with FreeSurfer's recon-all pipeline [19] and removing this section from the SUIT segmentation. With this procedure, the definition of the cerebellum was compatible with the external data used in this study. The test battery included data sets from three different samples. 30 datasets were from the same population as the training data and are hereafter referred to as EUH (Essen University Hospital). The second sample consisted of 20 data sets released as part of the ENIGMA MICCAI cerebellum challenge 2017 [20] and will be referred to hereafter as EMCC (voxel size = (1.0 × 1.0 × 1.2) mm^3^). This sample included T1w datasets of adolescent boys and girls, some healthy controls, and some individuals with attention deficit hyperactivity disorder (ADHD). Manual delineations of the cerebellum in this sample were provided by the Center for Neurodevelopmenta and Imaging Research at the Kennedy Krieger Institute (courtesy of Stewart Mostofsky). The third sample included 18 datasets obtained from the Internet Brain Segmentation Repository (IBSR) and will be referred to as IBSR in the following. These data consisted of modified (brain masked and intensity corrected) T1w 3D MRI datasets with voxel size of (0.94 × 0.94 × 1.5) mm^3^ and manually segmented cerebellum labels [21]. An overview of the different samples is given in Table 1.

**Table 1:** Overview of the three samples, including image orientation, number of subjects per sample and origin of ground truth labels. There are three variants for image orientation in the EUH sample, while it is consistent for both external samples (EMCC, IBSR). In total, there are 5 different image orientations in the data, namely left-posterior-superior (LPS), posterior-superior-left (PSL), posterior-superior-right (PSR), left-inferior- posterior (LIP), left-superior-anterior (LSA).

	EUH	EMCC	IBSR
image orientation	LPS(N_train_ = 25,N_test_ = 5), PSL(N_train_ = 26,N_test_ = 4), PSR(N_train_ = 137,N_test_ = 21)	LIP(N_test_ = 20)	LIP(N_test_ = 18)
ground truth label	manual + Freesurfer (Fischl, 2012)	manual	manual

All MRI data sets used for training and testing were denoised using a spatially adaptive non-local means algorithm [22], corrected for spatially low-frequency intensity variations using the N3 approach [23], and resampled to a voxel size of (1 × 1 × 1) mm^3^ using trilinear interpolation. All data sets were normalized volume-wise to have zero mean and unit variance.

**Convolutional neural network** – The network used in this application is based on a previously described two-pathway, fully-convolutional 3D neural network, built and optimized for segmentation of brain lesions and tumors, and known as DeepMedic [24]. The specific adapted version of this network and the training procedure used in this study were previously described by Jacobsen et al. [25]. In the present analysis, two augmentation techniques were used that employed changes in image intensity and image object orientation, respectively. Both augmentation techniques were implemented as part of the network training procedure. Five different sets of network weights were trained based on different combinations of the augmentation techniques. An overview of the trained networks is given in Table 2.

**Table 2:** Overview of the trained CNNs and the performed sensitivity analyses. A total of five networks were trained using different combinations of data augmentation. The sensitivity analysis was performed for all networks, but the parameter spaces differ depending on the augmentation technique.

	no rotation augmentation	rotation augmentation 1	rotation augmentation 2
no intensity augmentation	CNN_noaug_	CNN_rotaug_	CNN_rotaug2_
intensity augmentation	CNN_intaug_	CNN_rotaug+intaug_	–

**Rotation augmentation** – Two types of rotational augmentation were applied separately to achieve improved robustness with respect to spatial 3D object orientation in the data. Type 1 (rotaug) rotational augmentation involved mirroring the data with a probability of 50% with respect to all image axes (x, y, z). Type 2 (rotaug2) involved mirroring as in type 1 (rotaug), but also allowed random rotations of 0°, 30° or 50° about the x-axis. The x-axis was chosen because natural rotational movements by the subjects occur mainly around the horizontal x-axis (head nodding) and much less around the y- and z-axes.

**Intensity augmentation** – Intensity augmentation was based on a histogram analysis of the intensity distribution of the individual data sets. For each individual histogram, landmarks (positioned between 12.5% and 87.5% in steps of 12.5%) were determined. Then, the three independent parameters ($C_{1...3}$) of a second-degree polynomial, used as a transfer function to transform the histogram landmarks of one dataset to another dataset, were determined. By comparing all possible dataset pairs, the distributions for each of the parameters $C_{1...3}$ were determined and parameterized by a fit to a normal distribution. Using these empirically determined parameters (mean value and standard deviation for the distributions of $C_{1...3}$), the original intensities of the MRI data sets were modified during the training phase according to the following equation

(2) $$I^{\prime }={\tilde{C}}_{1}I^{2}+{\tilde{C}}_{2}I+{\tilde{C}}_{3}$$

where *I* denotes the original intensity and *I′* the modified intensity. The coefficients ${\tilde{C}}_{1...3}$ were randomly drawn from the previously determined normal distributions.

**Sensitivity analysis: parameter space selection** – Sensitivity analysis was used to determine the CNN performance resulting from the application of intensity augmentation, rotation augmentation and a combination of both, and to visualize the resulting patterns. The analyses were performed for the two different parameter spaces with respect to the orientation of the input image and the intensity range, respectively. To assess the performance sensitivity of the CNN with respect to image reorientation before and after the application of rotation augmentation, each test data set was systematically rotated around the x- and y-axes in the entire angular space [−180:20:180]°, using the center of the respective MRI data volume as the rotation center. This resulted in 19 × 19 (∑ = 361) systematic changes for each test dataset. The results were visualized as 2D heat maps that displayed the DSC as a function of the rotation angles around the x- and y-axis. The analysis was performed for all CNNs that used rotational augmentation and for the baseline CNN without augmentation (see Table 2) for 11 subjects from each test group (EUH, EMCC, IBSR). To investigate the sensitivity of the networks trained with and without intensity augmentation to intensity changes of the test data sets, their intensities were subjected to a linear transformation. This transformation modified the voxel intensities of each test data set by first multiplying the voxel values with a scale factor in the range between 1/5 and 5 in non-linear steps (1/5 1/(4.5) 1/4 1/(3.5) 1/3 1/2.5 1/2 1/(1.5) 1.0 1.5 2.0 2.5 3.0 3.5 4.0 4.5 5.0) before adding an offset in the range between -3.0 and 3.0 in linear steps of 3/8 (intensity offset). This resulted in 17 × 17 (∑ = 289) systematic variations per test data set. The Dice score (DSC) between the CNN segmentation of each manipulated data set and the ground truth segmentation was visualized in 2D heat maps as a function of intensity shift and scaling. This analysis was performed for all CNNs that used intensity augmentation and for the baseline CNNs without augmentation (see Table 2) for 14 subjects from each test group (EUH, EMCC, IBSR).

### Application case 2: segmentation of white matter tracts

**Data acquisition and preprocessing** – The MRI diffusion data used for training the neural network (TractSeg) [26] comprised 105 data sets from the Human Connectome Project (HCP) [27] acquired on a 3T Connectome MR scanner (Siemens Healthineers, Erlangen, Germany) with isotropic spatial resolution of 1.5 mm and 512 diffusion directions distributed over 5 different shells (b = 0; 1000; 3000; 5000; 10,000 s/mm^2^). As described in [26], the training data sets had been greatly augmented to improve generalizability. Two different versions of the pre-trained weights were used for the sensitivity analysis in this study. The first version, V.1.0.0, was delivered with the first version of TractSeg [28]. The second version of the weights, V1.1.0, [29] is the most current version of this HCP data set and was published with TractSeg version 2.1 [30]. In contrast to V.1.0.0, the network weights of V1.1.0 were obtained by randomly rotating the training data in the range of -45° to 45° around the x-, y- and z-axis in the data augmentation process. Diffusion-weighted test data from seven healthy volunteers were used to perform the sensitivity analysis using the software TractSeg [26]. The data were acquired in a multi-center study on 3T scanners (Magnetom Prisma, Siemens Healthineers, Erlangen Germany) using a 20-channel combined head-neck-coil and a multi-slice diffusion weighted imaging sequence [31]. Diffusion weightings were applied in 4 shells (b = 0; 830; 1660; 2490 s/mm^2^) and different unique directions (dirs) and, in case of b = 0, interlaced repetitions (reps) (N1 = 8 reps, N2 = 16 dirs, N3 = 32 dirs, N4 = 48 dirs). The sequence was acquired twice with opposite phase-encoding sign along the anterior-posterior (AP) direction, resulting in a total of 208 2D slice stacks. The spatial resolution was 1.5 mm isotropic. Each of the seven data sets was first denoised [32], then the two phase-encoding configurations of the b = 0 volumes in each subject data set were used to estimate individual susceptibility-induced distortions [33] and corrected in combination with eddy current and motion compensation [34]. As a prerequisite for the application of TractSeg, the fractional anisotropy (FA) map of each subject was used to estimate the rigid transformation into the MNI space. This transformation was then applied to the preprocessed diffusion-weighted data and the diffusion-weighting direction scheme was modified accordingly. The three diffusion directions corresponding to the largest spatial diffusivity amplitudes (direction peaks), as expected input from the TractSeg software, were extracted from the data using multi-shell multi-tissue constrained spherical deconvolution (CSD) and subsequent extraction of peaks using the software Mrtrix [35].

**Convolutional neural network** – The neural network used in this second application case was published by [26] and consists of a 2D encoder-decoder fully connected network architecture. The network segments whole-brain diffusion-weighted data into 72 different white matter tracts. The input to this network is a stack of 2D images with 9 channels, where the 9 channels correspond to the (x,y,z)-parameters of each of the three main diffusion directions (peaks) [26]. As mentioned before, the used pre-trained weights had been estimated by applying different types of data augmentations, including elastic deformation, spatial displacement and scaling, addition of Gaussian noise, as well as contrast and brightness modifications [26].

**Sensitivity analysis: parameter space selection** – Following transformation of all test data sets into the MNI space, a parameter space for rotations around the x- and y-axes was established, ranging from −16° to 16° in 2° steps, resulting in 17 × 17 (∑ = 289) different configurations. The center of rotation was defined individually for each subject as the center-of-mass of the intracranial brain volume. The selected angular range was chosen on the basis of preliminary experiments for rotations around the x- and the y-axis in the range from −180° to 180° without combination (i.e., either x- or y-rotation). The segmentation performance measured by DSC decreased rapidly for rotation angles > abs(±20°), which was confirmed by visual inspection, suggesting the ±16° range of interest. The diffusion-weighted data sets were spatially transformed using trilinear interpolation and the directional diffusion-weighting scheme needed for the reconstruction of the peaks was again modified accordingly. Direct rotation of the constrained spherical convolution (CSD) peak data sets was discarded to avoid vector interpolation between adjacent voxels. The ground truth segmentation of the 72 different white matter tracts was estimated for each subject using the 289 resulting segmentations in combination with the Simultaneous Truth and Performance Level Estimation (STAPLE) algorithm [36]. This algorithm was also recently used to generate consensus masks between raters in a tractography study [37]. Segmentations for the 289 different rotation combinations were compared for each subject and all white matter tracts against the estimated ground truth segmentation using DSC. The DSCs were plotted as a function of the x- and y-rotation angles as averages across all 72 white matter tracts for each test subject individually, as averages across test subjects for each white matter tract, and for a selection of white matter tract pairs (left + right) for all seven subjects individually. The entire procedure was performed using the two different versions of network weights [28], [29].

## Results

This section presents the results of the sensitivity analyses for both application cases.

### Application case 1: segmentation of the cerebellum

**Figure 2:**
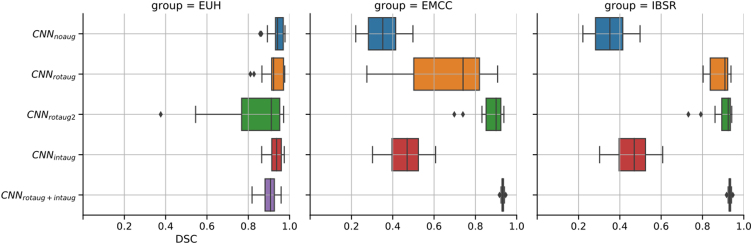
Horizontal box plots of DSC scores for CNN segmentation using the five trained networks without data augmentation (CNN_noaug_) and the applied four data augmentation methods (*CNN_rotaug_*;*CNN_rotaug2_*;*CNN_intaug_*;*CNN_rotaug+intaug_*), presented separately for all three studied test samples (*EUH*, *EMCC*, *IBSR*). For these plots, only the DSC values derived from test data without additional data manipulation (rotation or intensity manipulation) were used.

**Figure 3:**
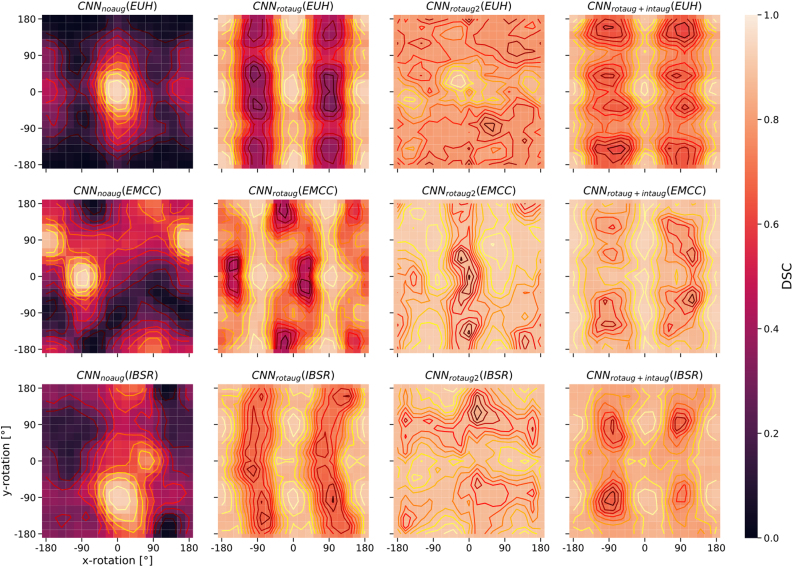
Sensitivity maps illustrating the CNN segmentation performance averaged over 11 test data sets from each test sample (rows: *EUH*, *EMCC*, *IBSR*) as a 2D function of x and y image rotations. The columns represent the four differently trained networks (columns: CNN_noaug_, CNN_rotaug_, CNN_rotaug2_, CNN_rotaug+intaug_).

**Figure 4:**
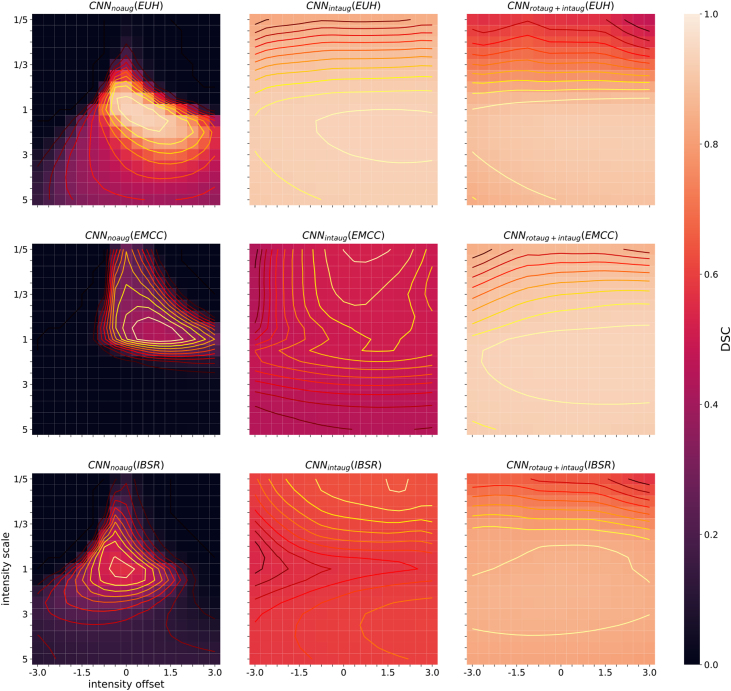
Sensitivity maps of CNN segmentation performance averaged over 14 test data sets from each test sample (rows: EUH, EMCC, IBSR) as a 2D function of image intensity offset and scale factor. The columns represent the three differently trained networks (columns: *CNN_noaug_*, *CNN_rotaug_*, *CNN_rotaug+intaug_*).

**Figure 5:**
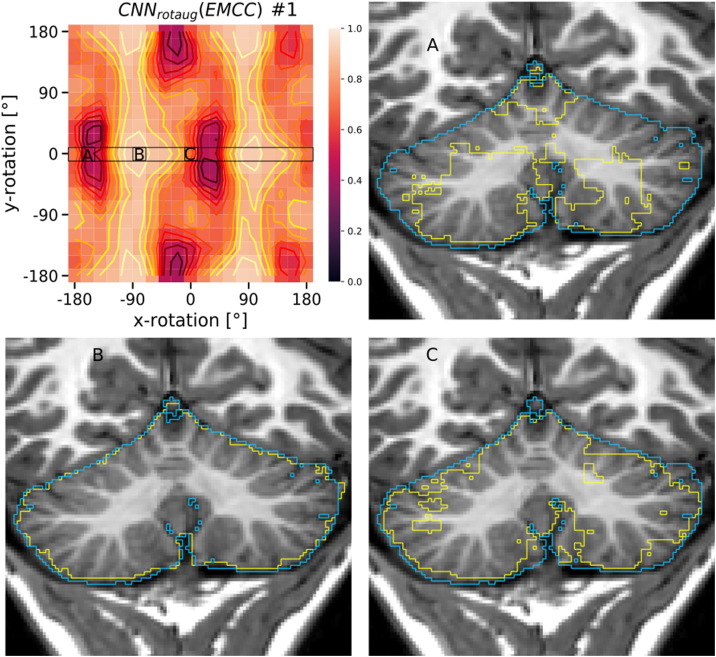
Sensitivity map *CNN_rotaug_*(*EMCC*) (taken from Fig. 3) and exemplary coronal slices of subject #1 from the EMCC test sample, with the ground truth label (blue) and a good (DSC = 0.94, (B)), bad (DSC = 0.42, (A)) and an intermediate DSC = 0.74, (C)) performing *CNN_rotaug_* segmentation (yellow) superimposed on the image. Mean DSC for the sensitivity map was 0.80 ± 0.18.

Figure 2 summarizes the performance results for the different augmentation methods (including no augmentation applied) for the three studied test populations (EUH, EMCC, IBSR). For the EUH group, no improvement was observed by data augmentation. In fact, with extended data rotation (rotaug2), the results worsened significantly. For the EMCC and IBSR groups, data augmentation and especially the rotation methods applied in the training phase led to significantly improved segmentation results. With intensity augmentation alone, only minor improvements were seen. The best results were obtained by combining rotation and intensity augmentation in the training phase. Fig. 3 contains visualizations of the sensitivity analysis regarding the effect of manipulating rotation in the test data sets on CNN segmentation performance. The sensitivity maps visualize the DSC values averaged over 11 test datasets from each test sample (EUH, EMCC, IBSR). Bright areas in the maps reflect good segmentation results for the corresponding set of parameters. For the network without data augmentation (CNN_noaug_, Fig. 3, first column), the bright spots on the maps allow us to draw conclusions about the positional relationship of the test data sets with respect to the training data. Especially for the EMCC and IBSR samples, suitable rotation parameters can be extracted from the maps to align the test datasets into the space of the training datasets (compare also Table 1). For example, the best segmentation performance for the IBSR sample appears around the parameters 0° on the x-axis and -90° on the y-axis. This transformation changes the orientation LSA (left-superior-anterior) – the orientation of the IBSR data set – into PSL (posterior-superior-left) orientation, which differs from PSR (posterior-superior-right) – the predominant orientation of the training data set – only by mirroring on the sagittal plane. Applying CNN_rotaug_, trained with random mirroring about all axes, to the rotationally manipulated test data sets resulted in symmetric patterns of the DSC distributions in the sensitivity maps (Fig. 3, second column). The strongly varying DSC values for CNN_noaug_ and CNN_rotaug_ across the parameter space are flatter with less pronounced maxima and minima for CNN_rotaug2_, which used rotation as well as mirroring in the training phase and achieved overall higher average segmentation scores. The maps for CNN_rotaug+inaug_ are similar to those of CNN_rotaug_, but with again overall higher performance scores. Figure 4 summarizes the results of an analogous sensitivity analysis to assess the effect of intensity manipulations in the test data sets on CNN segmentation performance. The subplots display the averaged DSC values as a function of the systematic changes in intensity offset and scale factor for all test subjects of the three test samples. CNN_noaug_ resulted in maps with the lowest averaged DSC values and the steepest descents in the selected parameter space (Fig. 4, left column). For the EMCC and IBSR data sets, the largest mean DSC values and flattest maps were obtained with CNN_rotaug+intaug_ when both intensity and rotation augmentation were used (except for EUH). For the EUH test population, CNN_intaug_ yielded the highest mean DSC values. To illustrate the resulting segmentation quality for different rotation configurations, Fig. 5 shows the realigned segmentations on a single coronal slice obtained for three different rotation manipulations around the x-axis compared to the ground truth for a single subject of the EMCC sample. The selected example in Fig. 5 demonstrates that in this case good segmentation (DSC = 0.94) can only be achieved with a rotation of about 90° around the x-axis. Without manipulation of the input data (x and y rotation = 0°) only a moderate segmentation result (DSC = 0.74) is obtained.

### Application case 2: segmentation of white matter tracts

**Figure 6:**
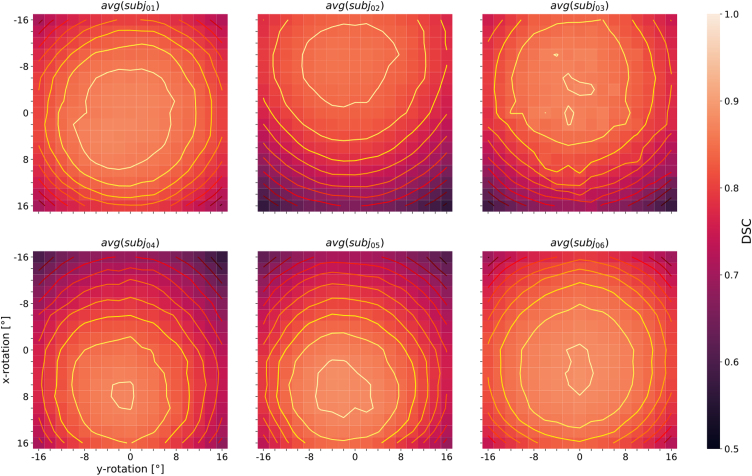
Heat maps of the DSC distribution with contour plots, resulting from the white matter tract segmentation as a function of input data rotations around the x- and y-axis (representing nodding and head wiggling), averaged across all white matter segments (n = 72) for 6 of the 7 subjects studied. The scaling of the color bar legend refers to the heat maps.

The effects of rotational manipulation of the test data sets on CNN segmentation performance of the white matter tracts was again assessed with DSC and plotted as two-dimensional distributions. The results averaged over all 72 white matter tracts are shown for 6 of the 7 subjects in Fig. 6. Besides the segmentation performance, the heat maps visualize the subject-specific orientation bias in relation to the training sample. The optimal rotational configuration was not observed for the standard configuration (0° x-rotation and 0° y-rotation), but was found off-center. Segmentation performance decreased with increasing distance from the maximum in all maps. The subject-specific contour lines are approximately symmetric about the y-axis (head wiggle axis), which can be explained by including several corresponding pairs of white matter tracts from the left and right brain hemisphere in the analysis and averaging over them. Since these tracts lead to more or less mirror-symmetric DSC patterns with respect to the y-axis upon rotational manipulation of the test data sets, the patterns become symmetric after averaging. With regard to the x-axis (head nodding axis), offsets of the contour patterns are observed, which can be explained by subject-specific anatomical features or suboptimal registration into the MNI space.

**Figure 7:**
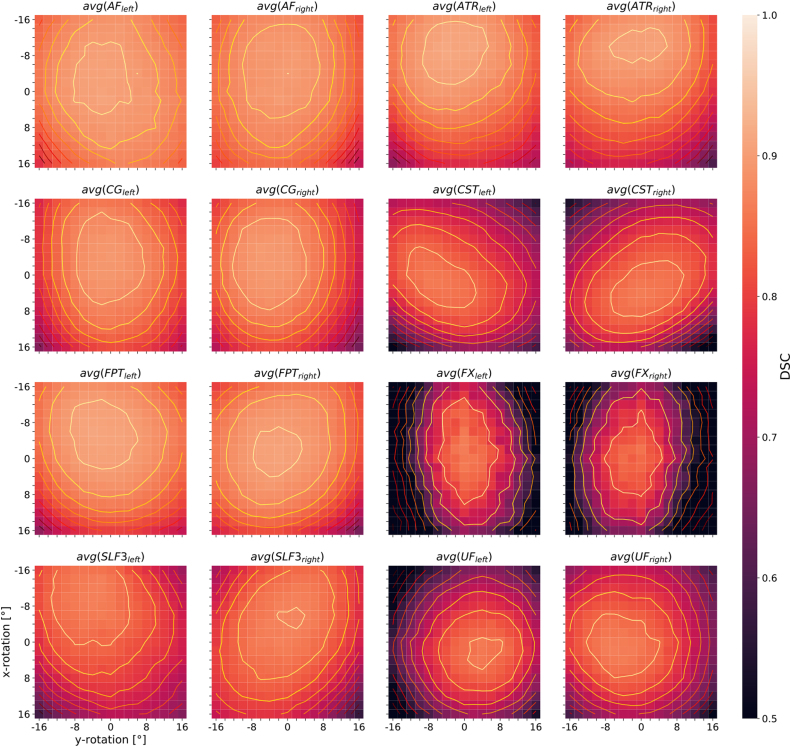
Heat maps of the DSC distribution with contour plots for several pairs of selected left- and right-sided white matter tracts (AF = arcuate fascicle, ATR = anterior thalamic radiation, CG = cingulum, CST = corticospinal tract, FPT = fronto-pontine tract, FX = fornix, SLF3 = superior longitudinal fascicle III, UF = uncinate fascicle). The DSC distribution is displayed as a function of input data rotation around the x- and y-axis (head nodding and wobbling), averaged across 7 subjects. The scaling of the color bar legend refers to the heat maps.

**Figure 8:**
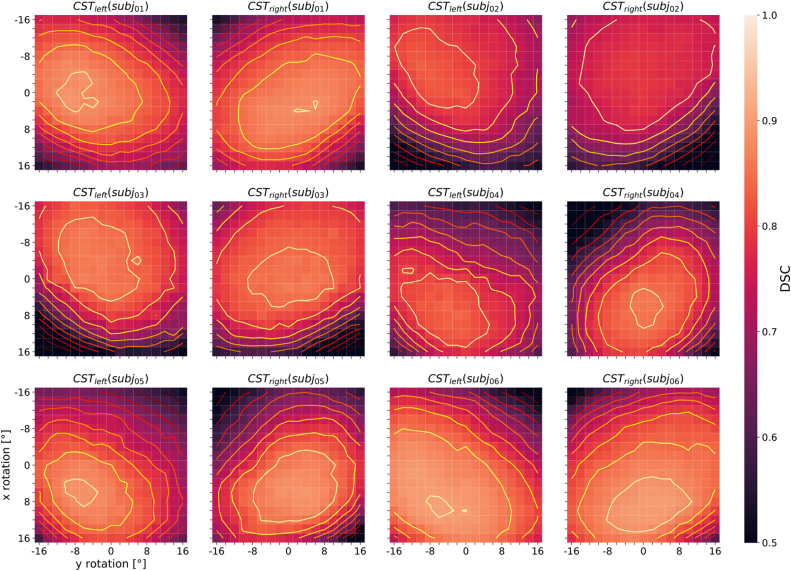
Representation of the segmentation performance of the corticospinal tract (CST), measured by DSC, as a function of the rotation of the input data around the x- and y-axis (head nodding and wobbling) for 6 of the 7 investigated subjects. The scaling of the color bar legend refers to the heat maps.

**Figure 9:**
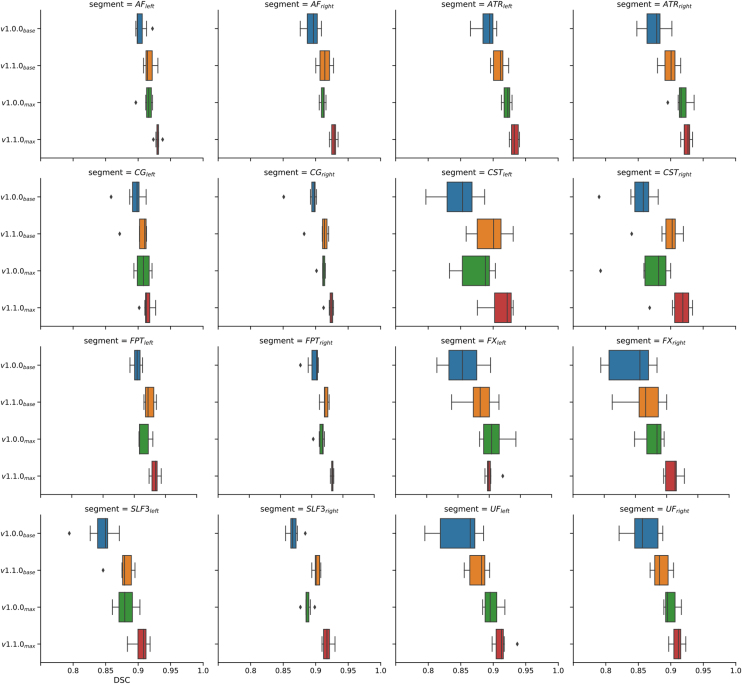
Boxplots of DSC values for a selected number of pairs of white matter tracts (same as in Figure 7), including AF, ATR, CG, CST, FPT, FX, SLF3, and UF. Each subplot shows the results for network weights of version V1.0.0 and V1.1.0 for the basic parameter configuration without artificial rotation as well as for the individual maxima in the investigated parameter space (x- and y-rotation).

Sensitivity maps were also created for individual white matter segments and then averaged across all seven subjects. A selection of the results for 8 left-sided and right-sided pairs of white matter tracts (AF = arcuate fascicle, ATR = anterior thalamic radiation, CG = cingulum, CST = corticospinal tract, FPT = fronto-pontine tract, FX = fornix, SLF3 = superior longitudinal fascicle III, UF = uncinate fascicle) is shown separately in Fig. 7. Similar to Fig. 6, these plots show decreasing segmentation performance with increasing distance from the center of the contour plots, which does not necessarily coincide with the center of the map. The white matter tract pairs generally lead to more or less strongly pronounced mirror-symmetric DSC-patterns between the corresponding left- and right-sided tract when the test data sets are rotationally manipulated and segmented, which can be seen particularly well for avg(CST_left_) and avg(CST_right_) in Fig. 7. Upon averaging, and because most of the 72 white matter segments considered by TractSeg are paired (except for the segments of the corpus callosum), axially symmetric patterns result as illustrated in Fig. 6. To demonstrate the consistency of the observed DSC patterns across subjects, the segmentation performance maps for one selected pair of white matter tracts (corticospinal tract) are presented individually for 6 individual subjects studied (Fig. 8). The mirror-symmetric pattern between the left and right tract with respect to y-rotation is observed in all subjects, whereas the bias for x-axis rotation differs among subjects. Additionally, Fig. 8 also shows the maximum position of each subject at different locations. E.g. for *subj*_*02*_ the maxima are shifted more to negative values along the y-axis (x rotation), which in turn is in accordance with the finding that *subj*_*02*_ has a general preference for better segmentation results towards negative x-rotation, which can be observed from Fig. 6. A similar observation can be made for *subj*_*04*_ and *subj*_*05*_, except that these have a preference for a positive x-rotation. In the case of *subj*_*01*_, *subj*_*03*_ and *subj*_*06*_, on the other hand, no correction of the x-rotation is required for optimal segmentation, as can be seen from both Fig. 6 and Fig. 8, since their maxima are close to the coordinate center (0,0). All segmentation analyses of the white-matter tracts were repeated with the second set of network weights (V1.1.0), which unlike the first version (V1.0.0) also included 3D data rotation in the data augmentation during training (±45° randomly around all three axes). With these weights, the patterns in the heat maps broadened in all analyses, which could be observed by averaging across all 72 tracts for 6 of the 7 subjects studied (data not shown). However, the drop in performance around the off-center optimum is similar to the results with version V1.0.0 of the network weights (compare Fig. 6). The maxima are in both cases located at similar positions and are subject-specific. To further demonstrate the general performance improvement with the new network weights, boxplots of the DSC values were generated for the same 8 pairs of white matter tracts as in Figure 7 (see Fig. 9). Each subplot contains the boxplots for the basic parameter configuration (i.e., x- and y-rotation angle = 0°) for both sets of network weights (i.e., versions V1.0.0 and V1.1.0) as well as for the corresponding maximum DSC values (i.e. optimal rotation angles). Improvement of segmentation performance was observed in all cases by using the network weight set V1.1.0 without any further manipulation of the input data. Further improvements were achieved, as expected, by using the optimal rotation angles. In some cases (e.g., tracts ATR, FX and UF, see Fig. 9) better segmentation results were obtained with network weights V1.0.0 and corresponding optimal rotation angles compared to the segmentation using version V1.1.0 and the basic parameter configuration.

## Discussion

In this study, we presented an approach to analyze the performance of deep neural networks. The analysis strategy was applied to two different application cases, each focused on the segmentation of complex brain structures. In case study one (segmentation of the cerebellum using DeepMedic), we investigated the effect of rotational and intensity augmentation of the training data sets and mapped the segmentation results of the differently trained CNNs applied to the appropriately modified test data sets in relation to the ground truth label (expressed by the DSC metric) as 2D functions over the corresponding parameter spaces. In case study two, we used a pre-trained network optimized for segmentation of white matter tracts based on diffusion-weighted data and examined the sensitivity of the network with respect to rotations of the test data sets. It was shown that data augmentation generally has a positive effect on segmentation performance. However, this general statement is only valid for samples that do not come from the training population. With data from the training population, the use of different augmentation methods can have even negative effects, as the results show. Thus, the successful integration of data augmentation depends mainly on the data to which the network will be applied later. The analysis also clearly showed the so-called training data set sampling bias [38], which was particularly evident in the rotation analysis in the first case study (cerebellar segmentation), but was also at least partially noticeable in the second case study (white matter tract segmentation using TractSeg). Without rotational augmentation, meaningful segmentation was achieved only when the test data sets had the same orientation as the training data sets, regardless of whether the latter originated from the same or from a different population. A more advanced approach to incorporate variations in training data more comprehensively is to use synthetic data generated using style transfer via GAN [13], [39]. However, this would be a process with unpredictable and thus uncontrollable effects and training data induced biases would become consolidated and more pronounced in an analysis like presented in this work. Generating synthetic data via style transfer for training and mixing it with real data, would only allow varying parameter, which are present in the training data, and thus the problem of training biases cannot really be solved in a targeted way.

It was also found that spatial transformation using simple data mirroring along the three spatial axes is not sufficient to compensate for the different orientation configurations and that different rotational augmentations can strongly affect the performance sensitivity maps (see groups EMCC and IBSR for CNN_rotaug_ and CNN_rotaug2_ in Fig. 3). Without such mappings, however, the effects of data augmentation cannot be adequately assessed. Although there are many studies [37], [40], [41], [42] that address the challenges of deep learning-based semantic segmentation, none of them has demonstrated such dependencies in detail. Our sensitivity mapping approach is easy to apply, but has some limitations. In the case of rotational analysis, the application is very simple because the parameter space to be investigated is clearly defined. In contrast, the manipulation of voxel intensities is much more complex. For the analysis in this study only a simple linear parameter manipulation was used, which is not able to map complex differences between intensity profiles of training data and test data to each other. Although the segmentation performance improved when applying intensity data augmentation using a more complicated method, it was also shown that the effects are not directly interpretable, especially when applied to new test samples. This effect was also described by Jacobsen et al. [25]. The intensity manipulation results are therefore difficult to use for network optimization. Despite this problem, however, we were able to show that the combination of these two augmentation techniques leads to an additive effect and thus improved segmentation performance compared to separate single augmentation methods. In principle, this sensitivity analysis can be performed for all conceivable parameters. In particular, the mapping of the network performance as a function of these parameters is suitable as a control tool when augmentation procedures are used. In the best case, a strategy for modifying the training procedure (e.g., by data augmentation) could be derived directly from the network architecture and the pre-trained-weights; however, such a strategy is currently not known. Although the application of the presented analysis requires a considerable number of calculations, these calculations are usually much more time efficient than training the network, so the presented approach is preferable to simply testing different augmentation strategies. This is especially true when the network architecture is not accessible. In such cases, the advantage of sensitivity analysis is that its results indicate how the test population data must be pre-processed to achieve optimal results. This preprocessing takes place in established programs for brain segmentation to an extensive extent [43] to avoid the problem that different parameterized input data lead to different results. Deep learning based approaches thus suffer from the same problems as classical methods, but are usually more robust and faster in processing. With case study two, we have shown that the analysis can also be performed even when no ground-truth is available. Here, in the absence of a reference, a consensus-building procedure (STAPLE, [36]) was used to compare the results of the different parameter configurations. However, when using this method, it should be noted that the calculated sensitivity maps are only qualitative in nature, as they are based on a reference defined for each parameter map individually. We would also like to point out that the observed effects may depend on the type of sampling within the processing. For example, small patches are usually applied to DL-based processing for technical implementation reasons, which can result in both advantages [44] and disadvantages. In the use cases shown here, DeepMedic used small 2D patches and TractSeg used large (144x144x9) patches. The advantages or disadvantages of using these patches will largely depend on the size ratio of the structure to be segmented and the size of the input samples. A sensitivity of the segmentation performance can be assumed based on the presented results of both patch-based (DeepMedic) and unpartitioned samples (TractSeg). Furthermore, it was found that reference generation using STAPLE only works, if there is a sufficiently high number of meaningful results in the set.

Another way of assessing segmentation performance could be done using relevance maps like in Lopatina et al. [45], where the relevance of true-positive and true-negative voxels is accumulated and used for a score [46]. However, it can be assumed that this will produce a score similar to the DICE score and thus, will not lead to a different conclusion. It should also be noted, that calculation of relevance maps is technically more complex and takes longer than, for example, the DICE score.

In both of our case studies, it was shown that there is a bias between the individual test data sets and the training population, which can be determined and quantified by finding the maximum in the corresponding sensitivity map. If this maximum is not at the position corresponding to a non-manipulated test data set, there is a bias between the test data set and the training population with respect to the parameter under investigation (e.g., rotation). If this maximum is located at scattered different positions for different test data sets, it might indicate suboptimal orientation alignment of individual test data sets with respect to the training population. If this maximum is consistently located in a narrowly defined spot on the map for the test data population, but not close to the center, this indicates a systematic deviation from the orientation of the training population. The latter effect, however, can be virtually eliminated by appropriate pre-processing of the data.

## Conclusion

A simple and straightforward method has been proposed to study the sensitivity of deep neural networks to the variability of input data in the context of semantic segmentation. The application of this method was demonstrated using two freely available networks. We were able to show how systematic changes in the input data affect segmentation performance, as well as the effects of different augmentation methods. The proposed approach supports both deep neural network developers to efficiently implement augmentation methods and users of such networks to optimally preprocess their test data.
