# Erratum to “Investigation of biases in convolutional neural networks for semantic segmentation using performance sensitivity analysis” [Z Med Phys 32 (2022) 346–360]

**DOI:** 10.1016/j.zemedi.2024.07.007

**Published:** 2024-09-02

**Authors:** Daniel Güllmar, Nina Jacobsen, Andreas Deistung, Dagmar Timmann, Stefan Ropele, Jürgen R. Reichenbach

**Affiliations:** a Medical Physics Group, Institute of Diagnostic and Interventional Radiology, Jena University Hospital - Friedrich Schiller University Jena, Germany; b University Clinic and Outpatient Clinic for Radiology, Department for Radiation Medicine, University Hospital Halle (Saale), Germany; c Department of Neurology, University Hospital Essen, University of Duisburg-Essen, Essen, Germany; d Department of Neurology, Karl-Franzens University of Graz, Austria; e Michael Stifel Center Jena for Data-Driven and Simulation Science, Friedrich-Schiller-University Jena, Jena, Germany

The publisher regrets that the declaration of competing interest statement was not included in the original article. The correct declaration of competing interest statement for this article is:

The authors declare that they have no known competing financial interests or personal relationships that could have appeared to influence the work reported in this paper.

The publisher would like to apologise for any inconvenience caused.

